# Conference Report: RADIONUCLIDE METROLOGY AND ITS APPLICATIONS—ICRM ’97 Gaithersburg, MD May 18–23, 1997

**DOI:** 10.6028/jres.102.041

**Published:** 1997

**Authors:** J. M. R. Hutchinson, Michael Unterweger

**Affiliations:** Ionizing Radiation Division, Physics Laboratory, National Institute of Standards and Technology, Gaithersburg, MD 20899-0001

## 1. Introduction

The International Committee for Radionuclide Metrology (ICRM) held its biennial conference at NIST on May 18–23, 1997, celebrating its 25th anniversary as the central international organization in radionuclide metrology. It was the first time that the conference has been held in this country.

The ICRM explicitly aims at being a forum for the dissemination of information on techniques, applications, and data related to the field of radionuclide metrology in order to advance international cooperation. Forty-two national and international scientific institutions are members, representing not only national metrology laboratories but international cooperative groups such as the International Bureau of Weights and Measures (BIPM), EURATOM, the International Atomic Energy Agency, and EUROMET (the metrology organization for the European community). The regions represented are from North and South America, Europe, Asia, Russia, and Africa.

The inspiration to form the ICRM derived from the need to bridge the gap between sophisticated metrological work in radioactivity and its applications at the user level. Increasingly, the realization of the potential for medical uses of radioactivity and the massive efforts to clean up the environment have called for international cooperation for which ICRM has been the major metrological vehicle.

The activities of ICRM are largely the responsibility of its working groups. At present there are seven such groups: α-particle spectrometry, γ- and β-ray spectrometry, life sciences, liquid scintillation, low-level measurement techniques, nuclear data, and radionuclide metrology techniques. Critical problems are investigated by these groups through specific actions, such as the improvement and categorization of all nuclear data relating to the nuclear fuel cycle, and many others. The conference helped focus the efforts of these groups and define needed future actions in the context of emerging technologies and rapidly changing needs.

Future concerns of the ICRM working groups include the development of liquid scintillation counting for the measurement of low-energy-β-particle emitting radionuclides, the development of mass spectrometry as a tool in the low-level measurement of long-lived low energy x- and β-particle emitters, the development of the digital recording system in coincidence counting, and the implementation of a uniform traceability system internationally as a means of utilizing the system of equivalence of radioactivity standards being developed under auspices of the BIPM.

## 2. Conference Summary

To accommodate the large number of papers without having parallel sessions, the papers were divided into oral presentations and posters. In addition to the 79 papers contained in the proceedings, there were approximately 15 other (non published) presentations made during the deliberations of the working groups. The following is a summary of the papers included in the proceedings.

### 2.1 Liquid Scintillation Counting

Liquid scintillation counting has become the most used method for direct measurement of activity in recent years and much fundamental work has been performed to establish its reliability. Nevertheless, there are important areas which need additional information to expand its capability and develop more effective methodologies. Some of the more important concerns were dealt with in the symposium including measurement of low-energy x- and β-emitters, Cerenkov counting, and the triple-to-double coincidence ratio and Centro de Investigaciones Energéticas, Medioambientales y Tecnólgicas (CIEMAT)/NIST methods.

For the low-energy β- and x-ray emitting radionuclides, ^55^Fe was calibrated by an efficiency calculation method using the γ-ray emitting ^54^Mn as a tracer from which the detection efficiency was calculated. ^49^V was standardized by the CIEMAT/NIST method which was studied for both organic and inorganic salts in four commercial scintillation cocktails. The massic activity of a solution of ^99^Tc was studied using ^60^Co as an efficiency tracing radionuclide. Measurements were made at the same time using conventional 4πβ liquid scintillation (LS) counting. Based on a comparison of these two methods, it was demonstrated that the β-spectral shape factor is a critical consideration when calculating the 4πβ efficiency. In the context of discrepancies in an international intercomparison of a ^63^Ni solution, the stability of sources in ultima gold liquid scintillation (LS) cocktail was investigated by varying Ni^++^ carrier concentration in the aqueous phase, HCl concentration in the aqueous phase, and aqueous mass fraction in the LS cocktail.

An improved Cerenkov standardization method was reported. A new expression for the Cerenkov counting efficiency was derived assuming that the Poisson law describes the generation of photoelectrons by the photo cathode. The radioactivity measurement of the short lived radiopharmaceutical ^188^Re by Cerenkov counting was also reported. ^139^Ce was standardized by the triple-to-double coincidence ratio method. Since dominant interaction between both K x rays and γ rays for ^139^Ce and the LS cocktail is due to the Compton effect, it was necessary to develop a new detection efficiency calculation program in order to determine the spectrum of the Compton electrons in the scintillator.

### 2.2 Radiation Detectors

Work on radiation detectors has aimed at the improvement of the techniques for metrology work to meet new requirements for accuracy, energy resolution, increased sensitivity at low levels of radioactivity, and improvement of pulse rate analysis at high counting rates.

A silicon-lithium (SiLi) detector was calibrated employing synchrotron radiation in the 1 keV to 7 keV range. The calibration by means of a pure light source, rather than calibrated low-energy x or y rays, allowed the processing of low-energy x-ray spectra with improved knowledge of the SiLi response, thus revealing the importance of secondary phenomena such as radiative Auger emission and satellite lines. Two papers discussed the use of an imaging plate in the measurement of radioactivity. One examined plutonium contaminated soil for hot particles and showed that they also contained silicon and iron. The other sought to decrease the measurement uncertainty (in this case by 10 % to 2 %) of the method by repetitive measurements of the same exposure. Three papers dealt with improvements in pulse processing. An instrument was developed to measure the interval-time distribution of nuclear detector signals. The disintegration rate of a source was obtained from an exponential fit through the intervaltime distribution. A second paper described the evaluation of counting losses due to small pulses in a windowless proportional counter. Another paper described a technique for the automatic compensation of dead time effects by artificially generating lost pulses and automatically adding them to the wave train. The bolometer is a technique which has great potential for the accurate measurement of low-energy photons due to two major advantages over competing devices: (i) the ultimate energy resolution is 100 times better than those obtained with conventional detectors and (ii) there is no window or dead layer effect. A paper described work with such a device in the temperature range of 30 mK using sources in the 5 keV to 6 MeV range. For the measurement of low-energy β-particle emitters in a windowless, gas-flow proportional counter, control of the pressure is critical because the high voltage plateaux are very sensitive to the gas density. A paper described a gain stabilization technique which was achieved by a two-stage backpressure regulation system that controls the pressure between 0 MPa and 6 MPa. Finally, in this group, an improved version of the phoswich detector was described. Various scintillators and optical filters were tested and the characteristics of systems using them are reported.

### 2.3 Counting Techniques

This section includes new developments in what is arguably the basic mission of the radionuclide metrologist—to provide archival calibrations of radionuclides and the methodologies by which this may be achieved, including new techniques.

One of the most important new developments in recent years, variously called a digital or pulse recording system, was described in three papers. The system continuously samples and stores pulse trains from radiation detectors, categorizing the individual pulses and marking them in time. Coincidence or anticoincidence analysis can be performed upon the stored information using appropiately designed computer software programs. Results for ^54^Mn, ^57^Co, and ^85^Sr were described in two of these papers. Data acquisition time can be reduced by at least an order of magnitude. This method is particularly advantageous when it is not possible or practical to make repeated counts on the same source due to very short half life or due to very low source activity.

A number of radionuclide calibrations were described as follows:

- ^56^Co: source preparation and disintegration rate measurement for the efficiency calibration of γ ray detectors
- ^110^mAg, ^75^Se, ^169^Yb: β efficiency extrapolation method
- ^222^Rn: measurement of the progenies with a Ge spectrometer
- ^67^Ga: 4πβ-y coincidence method
- ^198^Au (in gold foil): 4πβ-y coincidence method
- Noble gases: by means of solid reference sources, Monte Carlo calculations and previously calibrated ^85^Kr

It was demonstrated that correlations (or equivalent covariances) can play a major role in determining uncertainties in radioactivity counting. Examples were given. The photonuclear production of radionuclides with a 7 MeV to 32 MeV electron linac was described to produce neutron deficient nuclides by photonuclear reactions on targets of stable nuclei. New calibration figures for ^156^Sm, ^188^Re, ^233^Pa, and ^237^Np were reported for the NPL Secondary Standard Radionuclide Calibrator. Two papers described techniques for electronically processing input pulse trains from detectors that in the first case compared anticoincidence counting of low activities with normal background subtracted data, and secondly, subtraction of accidental coincidences and Compton scattered events by multi-channel time scaling techniques in γ-ray spectrometry.

### 2.4 Spectral Analysis

Analysis of α-, y-, and x-ray spectra has always been an inexact science because the physical basis for the shape of the tails is undeveloped and the analytical description has to be assumed. In a previous ICRM symposium, an attempt was made to derive such a physical basis with some success for α particle spectra in solid state detectors. The present meeting did not report on further work along these lines but did explore fitting functions, uncertainties in spectral analyses and application of such analyses. Two papers dealt with the evaluation of pulse shaping to determine (i) pulse pileup distortion and its effect on experimental uncertainty, and (ii) the use of pulse shaping to discriminate between Cerenkov events and scintillation events in a liquid scintillator, improve energy resolution in Cd Te detectors, direction sensing of charged particles in proportional counters, and discrimination of wall effects in ^3^He proportional counters. Two papers dealt with α particle spectrum analysis—one evaluated a fitting function that described the tails and the other made estimates of analytical uncertainties. The uranium enrichment measurement by x- and γ-ray spectrometry was described. A computer routine called COSPAJ has been developed that can be used in low-level α- and γ-ray spectral measurements. Finally, a Monte Carlo code was developed and used for the analysis of 4πγ ionization chamber response in the energy region below 200 keV.

### 2.5 Source Preparation

This session was very short consisting of only five accepted papers, possibly because much of the efforts in source preparation are in the liquid scintillation counting area described earlier. Two papers described the development of membranes to perform specialized tasks in the measurement process—the first demonstrated conducting membranes which can be used in 4πβ-y coincidence counting experiments, and the second uses a semi-permeable membrane to prevent the co-deposition of platinum eroded from the anode onto the stainless steel planchet thus reducing the source thickness. The other papers can be generally characterized as dealing with the production of specialized source configurations. A new class of tracers and images based on all carbon cage-like fullerences was described. Very small amounts of radioendofullerenes containing ^99m^Tc were produced and characterized. In another paper, the preparation of calibration standards and working standards from a high purity oxide solution using mass spectrometry was described. The radioactivity concentration was calculated using known half lives of the variously determined isotopic compositions. Finally, a technical procedure for preparing mixed, electroplated α sources of ^233^U, ^241^Am, and ^244^Cm in one instance and ^233^U, ^238^Pu, and ^244^Cm in another. The observed energy resolutions were 25 keV.

### 2.6 Low-level Measurements

Because low-level measurements, particularly environmental measurements of radioactivity, have large uncertainties and involve the *application* of metrological data to a specific area, they deviate from being classified under the heading of metrology in the classical sense. Nevertheless the ICRM and national metrology laboratories have invested considerable resources into this area because, without this investment, the transfer of national standards would be uncontrolled. This is because national standards are at very high activity levels compared to those in the environment and the many problems encountered in the analysis of radio-chemically complex matrices very often does not permit easy calibration with a national standard. One of the most effective methods for metrology transfer lies in the development of natural matrix standards. It is important that the community as a whole, including national metrology and environmental laboratories, participate in developing these carefully calibrated test materials, to ensure their acceptance and also to share the large expense. Three papers dealt with the development of natural matrix standards, two of which described new standards, namely an intertidal sediment from the Cumbrian coastline and a bone ash Standard Reference Material. The other presented new information concerning the chemical speciation of the radionuclidic content in an ocean sediment. A standard protocol for the identification of geochemical fractions of radioactive elements was a first step. Several papers reported on environmental measurements using new techniques to characterize radionuclides of interest in particular matrices as follows:

- ^93^mNb and ^94^Nb: low-level radioactive wastes
- ^243^Cm/^244^Cm ratio: environmental samples exposed to discharges from the nuclear fuel cycle using α spectrometry and spectral deconvolution
- ^14^Co_2_: environmental airborne carbon dioxide using passive sampling
- ^90^Sr: in bone ash.

A further paper describes the optimization of detection limits for the determination of the Sr/Ca mass ratio in a human bone specimen using fast neutron activation analysis.

### 2.7 Nuclear Data

The acquisition of accurate nuclear data is, of course, fundamental, not only to the development of archival calibrations but also to their use. The development of a calibration curve for a Ge detector depends critically on γ-ray abundances. In the liquid scintillation measurement of, say α- or β-particle emitters, the knowledge of delayed states or the characteristics of possible impurities is vital. The nuclear data sessions were comprised of reports of recently measured nuclear data of listed radionuclides given below with the reported work that was performed:

- ^244^Cm: α particle emission probabilities
- ^233^Th, ^233^Pa: half life measurements
- ^238^U decay chain: resolution of observed anomalies in the measured secular equilibrium between ^234^Th and ^234^mPa
- ^153^Sm: γ-ray emission probabilities
- ^125^Sb: determination of multipolarities and mixing ratios of the main γ transitions following ^125^Sb decay by means of γ-γ angular correlation
- 28 electron capture transitions: calculations of fractional electron capture probabilities
- ^89^Sr: measurement of the 909-12 keV y-ray emission probability
- ^55^Fe: half life measurement and standardization
- Radionuclides in the 24<Z<30 region: K_β_/K_y_ intensity ratios
- ^126^I: γ-ray probability per decay
- ^125^mTe to ^125^Sb: branching ratio
- ^182^Ta: γ-ray-emission probabilities
- ^222^Rn and daughters: emission probabilities of the main γ rays
- Eu radioisotopes: half life measurements

In addition, an assessment and evaluation of decay data for nuclear reactor applications were performed.

### 2.8 Intercomparisons

Intercomparisons are critical at the international level in establishing accepted national standards and at the national or lower level, as a means for disseminating these standards. The two sessions devoted to intercomparisons reported on these two aspects, separately.

At the international level, all reported intercomparisons were performed under the auspices of either the BIPM or the EUROMET. The BIPM sponsors the International Reference System (SIR) which records relative ionization chamber response to national γ-ray standards’ samples per Bq. A trial comparison of a solution of ^192^Ir was reported. The SIR system is being extended to α-and β-particle emitting radionuclides using LS counting as the basis. The first such intercomparison was performed with 10 laboratories submitting calibrated aqueous solutions of ^90^Sr to BIPM. Results showed a spread of the few tenths of one percent. The portability of the γ-ray SIR through the development of ionization chambers with configurations identical to the one at BIPM was discussed and found to be feasible.

In the framework of the EUROMET intercomparison program, ^55^Fe, ^63^Ni, tritiated water, and ^133^Xe were reported, the latter two using gas counting. The development of a Ge efficiency curve for 1 L Marinelli beakers containing material with density values ranging from 0.14 g L^−1^ to 1.7 g L^−1^ was reported.

At the national level reports were received from the U.K. (radionuclide calibrator intercomparisons in U.K. hospitals 1996, ^67^Ga and ^123^I); Canada (the Canadian experience in performing accuracy checks on administered doses of radiopharmaceuticals); Hungary (experience of 15 years on metrological supervision of radionuclide calibrators used in nuclear medicine); and Brazil (analysis of the Brazilian intercomparison program data—1991 to 1995—for radionuclide determination in environmental samples).

## 3. Future Conference

The next ICRM conference will be held in Prague, Czech Republic in 1999. The format is expected to be similar to the one presently reported.

## 4. For More Information

The Conference proceedings are being published as a special issue of Applied Radiation and Isotopes which is planned to come out in March 1998. To obtain more information about the next conference, or to be placed on the mailing list, write to: Pavel Dryak, CMI, Radiova 1, 102 00, Prague, CR Czech Republic, telephone: 420 2 67008244, fax: 420 2 67008466, email: prdyak@cmi.cz.

